# Hypodermic Injection of Ammonia for Opium Poisoning

**Published:** 1877-11

**Authors:** C. C. Fite


### HYPODERMIC INJECTION OF AMMONIA IN A CASE OF OPIUM POISONING.

Ou June the 19th, about 5 P. M., I was called to see a woman of about twenty-five years of age. She had, about 10 A. M., on that day, taken one and a half ounces of laudanum; but did not feel the full effect of it until the afternoon, when she lay down and covered her face with a handkerchief, saturated with chloroform.

When I reached her she was in a profound stupor; pulse feeble and intermitting, pupils contracted and stertorous respiration. I pursued the usual course of treatment; but as she grew steadily worse, I injected hypodermically one drachm of the aromat. spirits of ammonia, undiluted. In two minutes, she turned over in bed; her respiration became more natural, and all the other symptoms became mitigated. Nothing else was done for the case, but to give draughts of strong coffee every ten minutes. By 8 o'clock, the patient could talk intelligently, and recovered fully in twelve hours. There was an abscess formed at the point of injection, which gave some trouble for a few days. C. C. FITE, M. D., *in Medical Brief.*

